# Supplementary material for: Single Nucleotide Polymorphisms in the Vitamin D Metabolic Pathway as Survival Biomarkers in Colorectal Cancer
Source: Cancers (Basel). 2023 Aug 12;15(16):4077. doi: 10.3390/cancers15164077 (PMC10452893; doi:10.3390/cancers15164077)
Supplement: Supplementary file 1 [file cancers-15-04077-s001.zip › Table S1. Influence of clinical-pathological characteristics on overall survival of 127 CRC patients (1).pdf]

Table S1. Influence of clinical-pathological characteristics on overall survival of 127 CRC patients.

Independent variable	OS								
	N	Events	MST (mo)	$CI_{95\%}$	Log-rank p-value	Ref. cat	Univariate Cox Model		
							HR	$CI_{95\%}$	p-value
**Sex**									
**Female**	42	18	58.5	40.4-NR	0.500	-	-	-	-
**Male**	85	28	108.5	65.0-NR					
**Family history of cancer**									
**Yes**	64	19	134.5	62.3-NR	0.200	-	-	-	-
**No**	63	27	69.3	52.0-NR					
**Family history of CRC**									
**Yes**	27	6	134.5	NR-NR	**0.020**	Yes	2.731	1.153-6.467	0.022
**No**	100	40	62.1	48.9-NR					
**Smoking status**									
**Smokers**	27	8	NR	53.5-NR	0.090	Ex-smokers	1.039	0.434-2.488	0.931
**Ex-smokers**	44	14	114.9	78.4-NR			1	-	-
**Non-smokers**	56	24	48.9	36.3-NR			1.949	1.000-3.799	0.050
**Drinking status**									
**Drinkers**	24	7	134.5	75.9-NR	0.600	-	-	-	-
**Ex-drinkers**	7	2	NR	69.3-NR					
**Non-drinkers**	96	37	65.5	56.2-NR					
**Body mass index (BMI)**									
**<24**	27	9	56.2	39.6-NR	0.700	-	-	-	-
**>24**	100	37	108.5	62.3-NR					
**Type of CRC**									
**Colon**	89	33	108.5	62.3-NR	0.800	-	-	-	-
**Rectum**	38	13	62.1	41.9-NR					
**Age of CRC diagnosis**	127	-	-	-	**<0.001**	-	1.056	1.023-1.090	<0.001
**ECOG score**									
**0-1**	116	38	114.9	65.5-NR	**<0.001**	0-1	3.562	1.638-7.752	0.001
**2-4**	11	8	36.3	23.7-NR					
**Histopathology**									
**ADC**	107	39	114.9	62.1-NR	0.400	-	-	-	-
**Mucinous ADC**	20	7	52.1	25.8-NR					
**Tumor size**	127	-	-	-	0.300	-	-	-	-
**Lymph node involvement**									
**Yes**	92	33	65	52.0-NR	0.100	-	-	-	-
**No**	35	13	NR	69.3-NR					
**Number of lymph node involvement**									
**None**	35	13	NR	69.3-NR	**<0.001**	None	1	-	-
**1**	61	19	108.5	62.1-NR			1.224	0.599-2.501	0.579
**2**	31	14	30.6	23.2-NR			4.002	1.841-8.700	<0.001
~~**Histological grade**~~	~~127~~	~~-~~	~~-~~	~~-~~	~~0.500~~	~~-~~	~~-~~	~~-~~	~~-~~
**Histological grade**									
**Grade 1**	18	7	108.5	62.3-NR	0.600	-	-	-	-
**Grade 2**	94	34	78.4	58.5-NR					
**Grade 3**	15	5	61.9	23.7-NR					
**Metastasis**									
**Yes**	36	19	56.2	40.4-108.0	**0.001**	No	2.569	1.416-4.665	0.002
**No**	91	27	134.5	82.3-NR					
**Stage**									

I, II or IIIA	71	27	134.5	82.3-NR	**0.001**	I, II, IIIA	2.733	1.469- 5.087	0.002
IIIB, IV or IVB	56	19	52.1	36.3-NR					
**TNM stage**									
**I/II**	11	4	134.5	35.0-NR	0.900	-	-	-	-
**III/IV**	116	42	82.3	62.1-NR					
**Primary tumor resection**									
**Yes**	119	42	108.5	62.3-NR	0.100	-	-	-	-
**No**	8	4	20.5	15.3-NR					
**Adjuvant chemotherapy**									
**Fluoropyrimidines**	35	9	41.9	40.4-NR	**0.003**	Fluoropyrimidines + Oxaliplatin	2.059	0.407-1.776	0.076
**Fluoropyrimidines + Oxaliplatin**	70	24	134.5	108.5-NR			1	-	-
**Fluoropyrimidines + biologics drugs**	19	12	41.5	35.8-NR			3.123	0.358-3.177	0.001
**None**	3	1	30.2	NR-NR			6.554	1.053-1.786	0.074

ADC, adenocarcinoma. CRC, colorectal cancer. ECOG, Eastern Cooperative Oncology Group. HR, hazard ratio. $CI_{95\%}$, 95% confidence interval. MST, median survival time (months). NR, not reached. OS, Overall survival. TNM, Classification of Malignant Tumors.
