# Supplementary material for: Single Nucleotide Polymorphisms in the Vitamin D Metabolic Pathway as Survival Biomarkers in Colorectal Cancer
Source: Cancers (Basel). 2023 Aug 12;15(16):4077. doi: 10.3390/cancers15164077 (PMC10452893; doi:10.3390/cancers15164077)
Supplement: Supplementary file 1 [file cancers-15-04077-s001.zip › Table S10. Distribution of VDR haplotypes in overall survival in 127 CRC patients censored uncensored.pdf]

Table S10. Distribution of VDR haplotypes in overall survival in 127 CRC patients (censored/uncensored).

VDR Haplotypes	Haplotypes frequencies in Censored (%)	Haplotypes frequencies in Uncensored (%)
TCA	0.033802	0.052467
TAA	0.015951	0.045739
TAG	0.388519	0.358315
CCA	0.405105	0.295130
CCG	0.017883	0.011098
CAA	0.137735	0.215359
CAG	0.001006	0.021891
