# Supplementary material for: Single Nucleotide Polymorphisms in the Vitamin D Metabolic Pathway as Survival Biomarkers in Colorectal Cancer
Source: Cancers (Basel). 2023 Aug 12;15(16):4077. doi: 10.3390/cancers15164077 (PMC10452893; doi:10.3390/cancers15164077)
Supplement: Supplementary file 1 [file cancers-15-04077-s001.zip › Table S11. Distribution of CYP27B1 haplotypes in overall survival in 127 CRC patients censored uncensored.pdf]

Table S11. Distribution of *CYP27B1* haplotypes in overall survival in 127 CRC patients (censored/uncensored).

CYP27B1 Haplotype	Haplotype frequencies in Censored (%)	Haplotype frequencies in Uncensored (%)
CGGG	0.006191	0.000011
CGGT	0.290105	0.206511
CGAT	0.006173	0.000000
CAGT	0.006173	0.000000
GGGG	0.012346	0.000000
GAAG	0.666648	0.793467
GAAT	0.012364	0.000011
