# Supplementary material for: Single Nucleotide Polymorphisms in the Vitamin D Metabolic Pathway as Survival Biomarkers in Colorectal Cancer
Source: Cancers (Basel). 2023 Aug 12;15(16):4077. doi: 10.3390/cancers15164077 (PMC10452893; doi:10.3390/cancers15164077)
Supplement: Supplementary file 1 [file cancers-15-04077-s001.zip › Table S12. Associations between VDR haplotypes and overall survival in 127 CRC patients.pdf]

Table S12. Associations between VDR haplotypes and overall survival in 127 CRC patients.

VDR Haplotypes	Estimation	St Error	T-Test	HRR (IC95%)	p-value
TCA	-0.186	0.530	-0.352	0.829[0.293 - 2.347]	0.724
TAA	0.464	0.535	0.868	1.591 [0.557 - 4.548]	0.385
CCA	-0.240	0.240	-0.997	0.786 [0.490 - 1.260]	0.318
CCG	-0.573	1.022	-0.561	0.563 [0.075 - 4.176]	0.574
CAA	0.243	0.259	0.939	1.276 [0.767 - 2.123]	0.347
CAG	1.176	0.780	1.507	3.244 [0.702 - 14.980]	0.131

Ref. haplotype = TAG
-2x Log-likelihood (with covariates) = 366.533718
-2 x Log-likelihood (without covariates) =373.280064
(df = 6)
p-value =0.133
