# Supplementary material for: Single Nucleotide Polymorphisms in the Vitamin D Metabolic Pathway as Survival Biomarkers in Colorectal Cancer
Source: Cancers (Basel). 2023 Aug 12;15(16):4077. doi: 10.3390/cancers15164077 (PMC10452893; doi:10.3390/cancers15164077)
Supplement: Supplementary file 1 [file cancers-15-04077-s001.zip › Table S13. Influence of gene polymorphisms on progression-free survival of 127 CRC patients.pdf]

Table S13. Influence of gene polymorphisms on progression-free survival of 127 CRC patients.

Gen	SNPs	Genotype	PFS								
			N	Events	MST (mo)	$CI_{95\%}$	Log-rank p-value	Ref. cat.	Univariate Cox Model		
									HR	$CI_{95\%}$	p-value
VDR	**rs1544410 (BsmI)**	**TT**	33	16	73.3	37.0-NR	0.900	-	-	-	-
		**CT**	47	21	60.6	30.6-NR					
		**CC**	47	19	75.0	35.0-NR					
		**T**	80	37	73.3	38.6-NR	0.900	-	-	-	-
		**C**	94	40	63.3	38.1-NR	0.700		-	-	-
	**rs11568820 (Cdx2)**	**TT**	6	1	NR	16.7-NR	0.700	-	-	-	-
		**CT**	45	23	63.3	41.0-NR					
		**CC**	76	32	105.2	35.0-NR					
		**T**	51	24	63.3	41.0-NR	0.800	-	-	-	-
		**C**	121	55	63.3	41.0-NR	0.500	-	-	-	-
	**rs2228570 (FokI)**	**GG**	52	26	55.1	23.7-NR	**0.050**	AG	1.650	**0.934-2.916**	**0.085**
		**AG**	63	22	125.4	47.4-NR			1	-	-
		**AA**	12	8	19.8	6.23-NR			2.542	**1.126-5.742**	**0.025**
		**G**	115	48	75.0	43.8-NR	**0.070**	G	1.994	**0.939-4.232**	**0.072**
		**A**	75	30	105.2	41.0-NR	0.200	-	-	-	-
	**rs7975232 (ApaI)**	**AA**	48	27	43.8	34.5-NR	0.100	-	-	-	-
		**AC**	51	19	125.9	30.2-NR					
		**CC**	28	10	NR	51.1-NR					
		**A**	99	46	60.6	38.1-NR	0.200	-	-	-	-
		**C**	79	29	125.9	55.1-NR	**0.040**	C	1.732	**1.019-2.945**	**0.042**
	**rs731236 (TaqI)**	**GG**	27	15	41.0	18.6-NR	0.200	-	-	-	-
		**AG**	48	19	125.4	59.2-NR					
		**AA**	52	22	63.3	30.2-NR					
		**G**	75	34	73.3	43.8-NR	0.900	-	-	-	-
		**A**	100	41	105.0	51.1-NR	**0.090**	A	1.670	**0.916-3.046**	**0.094**
CYP27B1	**rs4646536**	**AA**	71	35	63.3	35.0-NR	0.800	-	-	-	-
		**AG**	42	15	105.2	37.0-NR					
		**GG**	14	6	NR	17.7-NR					
		**A**	113	50	73.3	41.0-NR	0.800	-	-	-	-
		**G**	56	21	105.2	38.6-NR	0.500	-	-	-	-
	**rs3782130**	**CC**	13	6	59.2	8.5-NR	0.700	-	-	-	-
		**CG**	43	15	105.2	38.6-NR					
		**GG**	71	35	63.3	35.0-NR					
		**C**	56	21	105.2	38.6-NR	0.400	-	-	-	-
		**G**	114	50	73.3	41.0-NR	0.900	-	-	-	-
	**rs10877012**	**TT**	12	6	59.2	8.5-NR	0.400	-	-	-	-
		**GT**	45	15	105.2	38.6-NR					
		**GG**	70	35	55.1	35.0-NR					
		**T**	57	21	105.2	38.6-NR	0.300	-	-	-	-
		**G**	115	50	73.3	41.0-NR	0.600	-	-	-	-
	**rs703842**	**GG**	14	6	NR	17.7-NR	0.800	-	-	-	-
		**AG**	42	15	105.2	37.0-NR					
		**AA**	71	35	63.3	35.0-NR					
		**G**	56	21	105.2	38.6-NR	0.500	-	-	-	-
		**A**	113	50	73.3	41.0-NR	0.800	-	-	-	-

CYP24A1	**rs6068816**	**CC**	89	39	63.3	43.8-NR	**0.010**	CT	1.241	0.619-2.489	0.543
		**CT**	28	10	NR	38.6-NR			1	-	-
		**TT**	10	7	14.2	3.1-NR			3.585	1.355-9.483	**0.010**
		**C**	117	49	75.0	47.4-NR	**0.004**	C	3.030	1.361-6.743	**0.006**
		**T**	38	17	75.0	19.8-NR	0.600	-	-	-	-
	**rs4809957**	**GG**	8	2	NR	34.5-NR	0.800	-	-	-	-
		**GA**	37	19	38.6	19.8-NR					
		**AA**	82	35	75.0	47.4-NR					
		**G**	45	21	51.1	23.7-NR	0.600	-	-	-	-
		**A**	119	54	63.3	41.0-NR	0.800	-	-	-	-
CYP2R1	**rs10741657**	**GG**	57	25	63.3	43.8-NR	1	-	-	-	-
		**AG**	56	23	125.4	35.0-NR					
		**AA**	14	8	60.6	18.6-NR					
		**G**	113	48	73.3	41.0-NR	0.900	-	-	-	-
		**A**	70	31	75.0	35.0-NR	1	-	-	-	-
GC	**rs7041**	**AA**	27	8	NR	60.6-NR	**0.004**	CC	1.149	0.440-2.997	0.777
		**AC**	71	39	38.1	18.5-73.3			2.742	1.321-5.691	**0.007**
		**CC**	29	9	125.9	105.8-NR			1	-	-
		**A**	98	47	55.1	37.0-NR	**0.030**	CC	2.226	1.083-4.574	**0.029**
		**C**	100	48	55.1	37.0-NR	0.100	-	-	-	-

HR, hazard ratio. $IC_{95\%}$, 95% confidence interval. MST, median survival time (months). NR, not reached.
