# Supplementary material for: Single Nucleotide Polymorphisms in the Vitamin D Metabolic Pathway as Survival Biomarkers in Colorectal Cancer
Source: Cancers (Basel). 2023 Aug 12;15(16):4077. doi: 10.3390/cancers15164077 (PMC10452893; doi:10.3390/cancers15164077)
Supplement: Supplementary file 1 [file cancers-15-04077-s001.zip › Table S14. Distribution of VDR haplotypes in progression free survival in 127 CRC patients censores uncensored.pdf]

Table S14. Distribution of VDR haplotypes in progression free survival in 127 CRC patients (censored/uncensored).

VDR Haplotypes	Haplotypes frequencies in Censored (%)	Haplotypes frequencies in Uncensored (%)
TCA	0.053268	0.023750
TAA	0.018345	0.039348
TAG	0.350923	0.410116
CCA	0.411077	0.312259
CCG	0.014528	0.012205
CAA	0.144070	0.187143
CAG	0.007789	0.015179
