# Supplementary material for: Single Nucleotide Polymorphisms in the Vitamin D Metabolic Pathway as Survival Biomarkers in Colorectal Cancer
Source: Cancers (Basel). 2023 Aug 12;15(16):4077. doi: 10.3390/cancers15164077 (PMC10452893; doi:10.3390/cancers15164077)
Supplement: Supplementary file 1 [file cancers-15-04077-s001.zip › Table S15. Distribution of CYP27B1 haplotypes in progression free survival in 127 CRC patients censored uncensored.pdf]

Table S15. Distribution of *CYP27B1* haplotypes in progression free survival in 127 CRC patients (censored/uncensored).

CYP27B1 Haplotype	Haplotype frequencies in Censored (%)	Haplotype frequencies in Uncensored (%)
CGGG	0.007063	0.000009
CGGT	0.274627	0.241062
CGAT	0.007042	0.000000
CAGT	0.007042	0.000000
GGGG	0.014085	0.000000
GAAG	0.683077	0.758920
GAAT	0.007063	0.000009
