# Supplementary material for: Single Nucleotide Polymorphisms in the Vitamin D Metabolic Pathway as Survival Biomarkers in Colorectal Cancer
Source: Cancers (Basel). 2023 Aug 12;15(16):4077. doi: 10.3390/cancers15164077 (PMC10452893; doi:10.3390/cancers15164077)
Supplement: Supplementary file 1 [file cancers-15-04077-s001.zip › Table S16. Associations between VDR haplotypes and progression free survival in 127 CRC patients.pdf]

Table S16. Associations between VDR haplotypes and progression free survival in 127 CRC patients.

VDR Haplotypes	Estimation	St Error	T-Test	HRR (IC95%)	p-value
TCA	-0.970	0.661	-1.466	0.378 [0.103 - 1.386]	0.142
TAA	0.032	0.543	0.060	1.033 [0.356 - 2.997]	0.951
CCA	-0.253	0.200	-1.262	0.775 [0.523 - 1.150]	0.206
CCG	-0.571	0.920	-0.621	0.564 [0.092 - 3.430]	0.534
CAA	0.068	0.239	0.286	1.071 [0.669 - 1.714]	0.774
CAG	0.037	0.852	0.043	1.038 [0.195 - 5.520]	0.964

Ref. haplotype = TAG
-2x Log-likelihood (with covariates) =460.739477
-2 x Log-likelihood (without covariates) = 466.690810
(df = 6)
p-value = 0.429
