# Supplementary material for: Single Nucleotide Polymorphisms in the Vitamin D Metabolic Pathway as Survival Biomarkers in Colorectal Cancer
Source: Cancers (Basel). 2023 Aug 12;15(16):4077. doi: 10.3390/cancers15164077 (PMC10452893; doi:10.3390/cancers15164077)
Supplement: Supplementary file 1 [file cancers-15-04077-s001.zip › Table S2. Influence of clinical-pathological characteristics on progression-free survival of 127 CRC patients (1).pdf]

Table S2. Influence of clinical-pathological characteristics on progression-free survival of 127 CRC patients.

Independent variable	PFS								
	N	Events	MST (mo)	$CI_{95\%}$	Log-rank p-value	Ref. cat	Univariate Cox Model		
							HR	$CI_{95\%}$	p-value
**Sex**									
**Female**	42	19	55.1	23.7-NA	0.900	-	-	-	-
**Male**	85	37	73.3	43.8-NA					
**Family history of cancer**									
**Yes**	64	25	105.2	41.0-NA	0.500	-	-	-	-
**No**	63	31	63.3	34.5-NA					
**Family history of CRC**									
**Yes**	27	8	125.9	60.6-NA	**0.080**	Yes	1.939	0.916-4.103	0.083
**No**	100	48	55.1	35.0-NA					
**Smoking status**									
**Smokers**	27	10	105.2	51.1-NA	0.400	-	-	-	-
**Ex-smokers**	44	19	125.4	60.6-NA					
**Non-smokers**	56	27	38.1	30.2-NA					
**Drinking status**									
**Drinkers**	24	9	125.9	73.3-NA	0.600	-	-	-	-
**Ex-drinkers**	7	2	NA	11.5-NA					
**Non-drinkers**	96	45	55.1	35.0-NA					
**Body mass index (BMI)**									
**<24**	27	11	55.1	14.2-NA	0.500	-	-	-	-
**>24**	100	45	75.0	41.0-NA					
**Type of CRC**									
**Colon**	89	38	105.2	47.4-NA	0.400	-	-	-	-
**Rectum**	38	18	43.8	23.7-NA					
**Age of CRC diagnosis**	127	-	-	-	**0.001**	-	1.051	1.021-1.081	0.001
**ECOG score**									
**0-1**	116	47	105.8	51.1-NA	**<0.001**	0-1	3.278	1.588-6.767	0.001
**2-4**	11	9	18.6	11.9-NA					
**Histopathology**									
**ADC**	107	48	73.3	38.6-NA	0.900	-	-	-	-
**Mucinous ADC**	20	8	60.6	18.6-NA					
**Tumor size**	127	-	-	-	0.200	-	-	-	-
**Lymph node involvement**									
**Yes**	92	44	41	27.3-NA	**0.010**	No	2.268	1.191-4.318	0.010
**No**	35	12	NA	75.0-NA					
**Number of lymph node involvement**									
**None**	35	12	NR	75.0-NR	**<0.001**	None	1	-	-
**1**	61	25	63.3	41.0-NR			1.779	0.819-3.552	0.102
**2**	31	19	18.6	17.7-73.3			3.752	1.787-7.876	<0.001
~~**Histological grade**~~	~~127~~	~~-~~	~~-~~	~~-~~	~~**0.060**~~	~~-~~	~~1.701~~	~~0.984-2.943~~	~~0.057~~
**Histological grade**									
**Grade 1**	18	6	105.8	60.6-NR	0.100	-	-	-	-
**Grade 2**	94	42	73.3	41.0-NR					
**Grade 3**	15	8	18.5	17.7-NR					
**Metastasis**									

**Yes**	36	30	19.8	14.2-59.2	**<0.001**	No	3.810	2.238-6.488	<0.001
**No**	91	26	125.9	125.9-NA					
**Stage**									
**I, II or IIIA**	71	24	125	125.4-NA	**<0.001**	I, II, IIIA	3.934	2.239-6.913	<0.001
**IIIB, IV or IVB**	56	32	35	17.7-60.6					
**TNM stage**									
**I/II**	11	4	125.9	43.8-NR	0.600	-	-	-	-
**III/IV**	116	52	63.3	38.6-NR					
**Primary tumor resection**									
**Yes**	119	49	105.2	51.1-NA	**0.008**	Yes	2.902	1.276-6.600	0.011
**No**	8	7	14.1	10.3-NA					
**Adjuvant chemotherapy**									
**Fluoropyrimidines**	35	15	38.1	18.6-NA	**<0.001**	Fluoropyrimidines + Oxaliplatin	2.545	0.342-2.734	0.006
**Fluoropyrimidines + Oxaliplatin**	70	24	125.4	105.8-NA			1	-	-
**Fluoropyrimidines + biologics drugs**	19	16	23.7	16.7-60.6			3.497	0.328-3.821	<0.001
**None**	3	1	NA	11.9-NA			3.124	1.039-1.096	0.273

ADC, adenocarcinoma. CRC, colorectal cancer. ECOG, Eastern Cooperative Oncology Group. HR, hazard ratio. $CI_{95\%}$, 95% confidence interval. MST, median survival time (months). NR, not reached. PFS, Progression-free survival. TNM, Classification of Malignant Tumors.
