# Supplementary material for: Single Nucleotide Polymorphisms in the Vitamin D Metabolic Pathway as Survival Biomarkers in Colorectal Cancer
Source: Cancers (Basel). 2023 Aug 12;15(16):4077. doi: 10.3390/cancers15164077 (PMC10452893; doi:10.3390/cancers15164077)
Supplement: Supplementary file 1 [file cancers-15-04077-s001.zip › Table S3. Minor allele frequencies of SNPs (1).pdf]

Table S3. Minor allele frequencies of SNPs.

Chr	SNP	Gen	Minor Allele	Major Allele	MAF
4	rs7041	*GC*	A	C	0.4921
11	rs10741657	*CYP2R1*	A	G	0.3307
12	rs1544410	*VDR*	T	C	0.4449
12	rs11568820	*VDR*	T	C	0.2244
12	rs2228570	*VDR*	A	G	0.3425
12	rs7975232	*VDR*	C	A	0.4213
12	rs731236	*VDR*	G	A	0.4016
12	rs3782130	*CYP27B1*	C	G	0.2717
12	rs4646536	*CYP27B1*	G	A	0.2756
12	rs703842	*CYP27B1*	G	A	0.2756
12	rs10877012	*CYP27B1*	T	G	0.2717
20	rs6068816	*CYP24A1*	T	C	0.1890
20	rs4809957	*CYP24A1*	G	A	0.2087

Chr, Chromosome. MAF, Minor allele frequency.
