# Supplementary material for: Single Nucleotide Polymorphisms in the Vitamin D Metabolic Pathway as Survival Biomarkers in Colorectal Cancer
Source: Cancers (Basel). 2023 Aug 12;15(16):4077. doi: 10.3390/cancers15164077 (PMC10452893; doi:10.3390/cancers15164077)
Supplement: Supplementary file 1 [file cancers-15-04077-s001.zip › Table S4. Hardy-Weinberg Equilibrium (OS) (1).pdf]

Table S4. Hardy-Weinberg Equilibrium (OS).

Chr	SNP	Sample	Gen	Minor Allele	Major Allele	Genotype counts	Observed heterozygosity	Expected heterozygosity	p-value
4	rs7041	All	*GC*	A	C	27/71/29	0.5591	0.4999	0.2169
4	rs7041	Success	*GC*	A	C	5/30/11	0.6522	0.4915	**0.0394**
4	rs7041	No Success	*GC*	A	C	22/41/18	0.5062	0.4988	1
11	rs10741657	All	*CYP2R1*	A	G	14/56/57	0.4409	0.4427	1
11	rs10741657	Success	*CYP2R1*	A	G	6/21/19	0.4565	0.4601	1
11	rs10741657	No Success	*CYP2R1*	A	G	8/35/38	0.4321	0.4314	1
12	rs1544410	All	*VDR*	T	C	33/47/47	0.3701	0.4939	**0.0067**
12	rs1544410	Success	*VDR*	T	C	12/18/16	0.3913	0.4962	0.1491
12	rs1544410	No Success	*VDR*	T	C	21/29/31	0.358	0.4924	**0.0140**
12	rs11568820	All	*VDR*	T	C	6/45/76	0.3543	0.3481	1
12	rs11568820	Success	*VDR*	T	C	0/21/25	0.4565	0.3523	0.0868
12	rs11568820	No Success	*VDR*	T	C	6/24/51	0.2963	0.3457	0.2021
12	rs2228570	All	*VDR*	A	G	12/63/52	0.4961	0.4504	0.3255
12	rs2228570	Success	*VDR*	A	G	3/23/20	0.500	0.4317	0.4926
12	rs2228570	No Success	*VDR*	A	G	9/40/32	0.4938	0.4597	0.6308
12	rs7975232	All	*VDR*	C	A	28/51/48	0.4016	0.4876	**0.0468**
12	rs7975232	Success	*VDR*	C	A	8/17/21	0.3696	0.4601	0.2031
12	rs7975232	No Success	*VDR*	C	A	20/34/27	0.4198	0.4963	0.1807
12	rs731236	All	*VDR*	G	A	27/48/52	0.3780	0.4806	**0.0167**
12	rs731236	Success	*VDR*	G	A	11/14/21	0.3043	0.4764	**0.0150**
12	rs731236	No Success	*VDR*	G	A	16/34/31	0.4198	0.4829	0.2530
12	rs3782130	All	*CYP27B1*	C	G	13/43/71	0.3386	0.3957	0.1159
12	rs3782130	Success	*CYP27B1*	C	G	04/11/1931	0.2391	0.3277	0.0743
12	rs3782130	No Success	*CYP27B1*	C	G	9/32/40	0.3951	0.4268	0.6013
12	rs4646536	All	*CYP27B1*	G	A	14/42/71	0.3307	0.3993	0.0727
12	rs4646536	Success	*CYP27B1*	G	A	04/11/1931	0.2391	0.3277	0.0743
12	rs4646536	No Success	*CYP27B1*	G	A	10/31/40	0.3827	0.4314	0.3091
12	rs703842	All	*CYP27B1*	G	A	14/42/71	0.3307	0.3993	0.0727
12	rs703842	Success	*CYP27B1*	G	A	04/11/1931	0.2391	0.3277	0.0743
12	rs703842	No Success	*CYP27B1*	G	A	10/31/40	0.3827	0.4314	0.3091
12	rs10877012	All	*CYP27B1*	T	G	12/45/70	0.3543	0.3957	0.2617
12	rs10877012	Success	*CYP27B1*	T	G	04/11/1931	0.2391	0.3277	0.0743
12	rs10877012	No Success	*CYP27B1*	T	G	8/34/39	0.4198	0.4268	1
20	rs6068816	All	*CYP24A1*	T	C	10/28/89	0.2205	0.3065	**0.0029**
20	rs6068816	Success	*CYP24A1*	T	C	06/10/1930	0.2174	0.3639	**0.0098**
20	rs6068816	No Success	*CYP24A1*	T	C	4/18/59	0.2222	0.2695	0.1114
20	rs4809957	All	*CYP24A1*	G	A	8/37/82	0.2913	0.3302	0.1826
20	rs4809957	Success	*CYP24A1*	G	A	2/14/30	0.3043	0.3147	1
20	rs4809957	No Success	*CYP24A1*	G	A	6/23/52	0.2840	0.3387	0.1831

Chr, chromosome.
