# Supplementary material for: Single Nucleotide Polymorphisms in the Vitamin D Metabolic Pathway as Survival Biomarkers in Colorectal Cancer
Source: Cancers (Basel). 2023 Aug 12;15(16):4077. doi: 10.3390/cancers15164077 (PMC10452893; doi:10.3390/cancers15164077)
Supplement: Supplementary file 1 [file cancers-15-04077-s001.zip › Table S5. Hardy-Weinberg Equilibrium (PFS) (1).pdf]

Table S5. Hardy-Weinberg Equilibrium (PFS).

Chr	SNP	Sample	Gen	Minor Allele	Major Allele	Genotype counts	Observed heterozygosity	Expected heterozygosity	p-value
4	rs7041	All	*GC*	A	C	27/71/29	0.5591	0.4999	0.2169
4	rs7041	Success	*GC*	A	C	8/39/9	0.6964	0.4998	**0.0068**
4	rs7041	No Success	*GC*	A	C	19/32/20	0.4507	0.4999	0.4759
11	rs10741657	All	*CYP2R1*	A	G	14/56/57	0.4409	0.4427	1
11	rs10741657	Success	*CYP2R1*	A	G	8/23/25	0.4107	0.4539	0.5554
11	rs10741657	No Success	*CYP2R1*	A	G	6/33/32	0.4648	0.4329	0.7833
12	rs1544410	All	*VDR*	T	C	33/47/47	0.3701	0.4939	**0.0067**
12	rs1544410	Success	*VDR*	T	C	16/21/19	0.375	0.4986	0.0649
12	rs1544410	No Success	*VDR*	T	C	17/26/28	0.3662	0.4880	**0.0499**
12	rs11568820	All	*VDR*	T	C	6/45/76	0.3543	0.3481	1
12	rs11568820	Success	*VDR*	T	C	1/23/32	0.4107	0.3468	0.2604
12	rs11568820	No Success	*VDR*	T	C	5/22/44	0.3099	0.3491	0.3203
12	rs2228570	All	*VDR*	A	G	12/63/52	0.4961	0.4504	0.3255
12	rs2228570	Success	*VDR*	A	G	8/22/26	0.3929	0.4483	0.3756
12	rs2228570	No Success	*VDR*	A	G	4/41/26	0.5775	0.4520	**0.0338**
12	rs7975232	All	*VDR*	C	A	28/51/48	0.4016	0.4876	**0.0468**
12	rs7975232	Success	*VDR*	C	A	10/19/27	0.3393	0.4539	0.0753
12	rs7975232	No Success	*VDR*	C	A	18/32/21	0.4507	0.4991	0.4756
12	rs731236	All	*VDR*	G	A	27/48/52	0.3780	0.4806	**0.0167**
12	rs731236	Success	*VDR*	G	A	15/19/22	0.3393	0.4922	**0.0283**
12	rs731236	No Success	*VDR*	G	A	12/29/30	0.4085	0.4679	0.3120
12	rs3782130	All	*CYP27B1*	C	G	13/43/71	0.3386	0.3957	0.1159
12	rs3782130	Success	*CYP27B1*	C	G	6/15/35	0.2679	0.3659	0.0614
12	rs3782130	No Success	*CYP27B1*	C	G	7/28/36	0.3944	0.4166	0.7749
12	rs4646536	All	*CYP27B1*	G	A	14/42/71	0.3307	0.3993	0.0727
12	rs4646536	Success	*CYP27B1*	G	A	6/15/35	0.2679	0.3659	0.0615
12	rs4646536	No Success	*CYP27B1*	G	A	8/27/36	0.3803	0.4222	0.4051
12	rs703842	All	*CYP27B1*	G	A	14/42/71	0.3307	0.3993	0.0727
12	rs703842	Success	*CYP27B1*	G	A	6/15/35	0.2679	0.3659	0.0615
12	rs703842	No Success	*CYP27B1*	G	A	8/27/36	0.3803	0.4222	0.4051
12	rs10877012	All	*CYP27B1*	T	G	12/45/70	0.3543	0.3957	0.2617
12	rs10877012	Success	*CYP27B1*	T	G	6/15/35	0.2679	0.3659	0.0615
12	rs10877012	No Success	*CYP27B1*	T	G	6/30/35	0.4225	0.4166	1
20	rs6068816	All	*CYP24A1*	T	C	10/28/89	0.2205	0.3065	**0.0029**
20	rs6068816	Success	*CYP24A1*	T	C	07/10/1939	0.1786	0.3367	**0.0012**
20	rs6068816	No Success	*CYP24A1*	T	C	3/18/50	0.2535	0.2809	0.3994
20	rs4809957	All	*CYP24A1*	G	A	8/37/82	0.2913	0.3302	0.1826
20	rs4809957	Success	*CYP24A1*	G	A	2/19/35	0.3393	0.3264	1
20	rs4809957	No Success	*CYP24A1*	G	A	6/18/47	0.2535	0.3333	0.0660

Chr, chromosome.
