# Supplementary material for: Single Nucleotide Polymorphisms in the Vitamin D Metabolic Pathway as Survival Biomarkers in Colorectal Cancer
Source: Cancers (Basel). 2023 Aug 12;15(16):4077. doi: 10.3390/cancers15164077 (PMC10452893; doi:10.3390/cancers15164077)
Supplement: Supplementary file 1 [file cancers-15-04077-s001.zip › Table S6. Linkage disequilibrium of SNPs (1).pdf]

Table S6. Linkage disequilibrium of SNPs.

**Chr**	**BP**	**SNP**	**Gen**	**Chr**	**BP**	**SNP**	**Gen**	**R^2**	**D’**
12	47846052	rs1544410	VDR	12	47845054	rs7975232	VDR	0.373517	0.782038
12	47846052	rs1544410	VDR	12	47844974	rs731236	*VDR*	0.732418	0.907370
12	47845054	rs7975232	VDR	12	47844974	rs731236	*VDR*	0.391021	0.916933
12	57768115	rs3782130	*CYP27B1*	12	57764205	rs4646536	*CYP27B1*	0.949729	0.979861
12	57768115	rs3782130	*CYP27B1*	12	57768956	rs703842	*CYP27B1*	0.949729	0.979861
12	57768115	rs3782130	*CYP27B1*	12	57768302	rs10877012	*CYP27B1*	0.965225	0.980033
12	57764205	rs4646536	*CYP27B1*	12	57768956	rs703842	*CYP27B1*	0.966623	0.980215
12	57764205	rs4646536	*CYP27B1*	12	57768302	rs10877012	*CYP27B1*	0.916071	0.959577
12	57768956	rs703842	*CYP27B1*	12	57768302	rs10877012	*CYP27B1*	0.916071	0.959577

Chr, Chromosome. BP, Physical position (base-pair).
