# Supplementary material for: Single Nucleotide Polymorphisms in the Vitamin D Metabolic Pathway as Survival Biomarkers in Colorectal Cancer
Source: Cancers (Basel). 2023 Aug 12;15(16):4077. doi: 10.3390/cancers15164077 (PMC10452893; doi:10.3390/cancers15164077)
Supplement: Supplementary file 1 [file cancers-15-04077-s001.zip › Table S7. Frequencies of the VDR haplotypes in 127 CRC patients.pdf]

Table S7. Frequencies of the *VDR* haplotypes in 127 CRC patients.

***VDR* rs1544410 (BsmI)**	***VDR* rs7975232 (ApaI)**	***VDR* rs731236 (TaqI)**	**Total**	**Cumulative frequency**
T	A	G	0.3766	0.3766
C	C	A	0.3706	0.7472
C	A	A	0.1633	0.9105
T	C	A	0.0369	0.9474
T	A	A	0.0277	0.9750
C	A	G	0.0112	0.9862
C	C	G	0.0100	0.9963
T	C	G	0.0037	1
