# Supplementary material for: Single Nucleotide Polymorphisms in the Vitamin D Metabolic Pathway as Survival Biomarkers in Colorectal Cancer
Source: Cancers (Basel). 2023 Aug 12;15(16):4077. doi: 10.3390/cancers15164077 (PMC10452893; doi:10.3390/cancers15164077)
Supplement: Supplementary file 1 [file cancers-15-04077-s001.zip › Table S8. Frequencies of the CYP27B1 haplotypes in 127 CRC patients.pdf]

Table S8. Frequencies of the *CYP27B1* haplotypes in 127 CRC patients.

CYP27B1 **rs3782130**	***CYP27B1*** **rs4646536**	***CYP27B1*** **rs703842**	***CYP27B1*** **rs10877012**	**Total**	**Cumulative frequency**
G	A	A	G	0.7165	0.7165
C	G	G	T	0.2598	0.9764
G	G	G	G	0.0079	0.9842
C	G	G	G	0.0040	0.9882
G	A	A	T	0.0040	0.9921
C	A	G	T	0.0039	0.9961
C	G	A	T	0.0039	1
