# Supplementary material for: Single Nucleotide Polymorphisms in the Vitamin D Metabolic Pathway as Survival Biomarkers in Colorectal Cancer
Source: Cancers (Basel). 2023 Aug 12;15(16):4077. doi: 10.3390/cancers15164077 (PMC10452893; doi:10.3390/cancers15164077)
Supplement: Supplementary file 1 [file cancers-15-04077-s001.zip › Table S9. Influence of gene polymorphisms on overall survival of 127 CRC patients.pdf]

Table S9. Influence of gene polymorphisms on overall survival of 127 CRC patients.

Gen	SNPs	Genotype	OS								
			N	Events	MST (mo)	$CI_{95\%}$	Log-rank p-value	Ref. cat	Univariate Cox Model		
									HR	$CI_{95\%}$	p-value
VDR	rs1544410 (BsmI)	**TT**	33	12	108.5	41.9-NR	0.800	-	-	-	-
		**CT**	47	18	62.3	40.3-NR					
		**CC**	47	16	82.3	65.0-NR					
		**T**	80	30	108.5	48.6-NR	0.600	-	-	-	-
		**C**	94	34	82.3	61.3	0.900	-	-	-	-
	rs11568820 (Cdx2)	**TT**	6	0	NR	NR-NR	0.300	-	-	-	-
		**CT**	45	21	65.5	48.9-NR					
		**CC**	76	25	108.5	62.1-NR					
		**T**	51	21	75.9	52.0-NR	0.400	-	-	-	-
		**C**	121	46	82.3	61.3-NR	0.200	-	-	-	-
	rs2228570 (FokI)	**GG**	52	20	75.9	58.5-NR	0.900	-	-	-	-
		**AG**	63	23	114.9	52.1-NR					
		**AA**	12	3	NR	78.4-NR					
		**G**	115	43	82.3	61.3-NR	0.800	-	-	-	-
		**A**	75	26	114.9	53.5-NR	0.700	-	-	-	-
	rs7975232 (ApaI)	**AA**	48	21	48.9	39.6-NR	**0.090**	CC	2.181	0.962-4.944	**0.062**
		**AC**	51	17	114.9	61.3-NR			1.268	0.542-2.964	0.584
		**CC**	28	8	NR	75.9-NR			1	-	-
		**A**	99	38	65.5	52.0-NR	0.200	-	-	-	-
		**C**	79	25	134.5	69.3-NR	**0.030**	C	1.187	1.039-3.771	**0.037**
	rs731236 (TaqI)	**GG**	27	11	41.9	36.3-NR	**0.090**	AG	2.409	0.818-3.271	**0.034**
		**AG**	48	14	134.5	62.3-NR			1	-	-
		**AA**	52	21	75.9	58.5-NR			1.635	1.067-5.436	**0.164**
		**G**	75	25	108.5	53.5-NR	0.500	-	-	-	-
		**A**	100	35	134.5	65.0-NR	**0.080**	A	1.825	0.915- 3.637	**0.087**
CYP27B1	rs4646536	**AA**	7	31	75.9	53.5-NR	0.600	-	-	-	-
		**AG**	42	11	NR	48.9-NR					
		**GG**	14	4	114.9	62.1-NR					
		**A**	113	42	78.4	58.5-NR	0.500	-	-	-	-
		**G**	56	15	NR	62.1-NR	0.300	-	-	-	-
	rs3782130	**CC**	13	4	114.9	62.1-NR	0.500	-	-	-	-
		**CG**	43	11	NR	48.9-NR					
		**GG**	71	31	75.9	53.5-NR					
		**C**	56	15	NR	62.1-NR	0.200	-	-	-	-
		**G**	114	42	78.4	58.5-NR	0.500	-	-	-	-
	rs10877012	**TT**	12	4	114.9	62.1-NR	0.400	-	-	-	-
		**GT**	45	11	NR	61.3-NR					
		**GG**	70	31	69.3	53.5-NR					
		**T**	57	15	NR	62.1-NR	0.200	-	-	-	-
		**G**	115	42	82.3	58.5-NR	0.800	-	-	-	-
	rs703842	**GG**	14	4	114.9	62.1-NR	0.600	-	-	-	-
		**AG**	42	11	NR	48.9-NR					

		**AA**	71	31	75.9	53.5-NR					
		**G**	56	15	114.9	62.1-NR	0.300	-	-	-	-
		**A**	113	42	78.4	58.5-NR	0.500	-	-	-	-
CYP24A1	**rs6068816**	**CC**	89	30	82.3	58.5-NR	0.200	-	-	-	-
		**CT**	28	10	114.9	40.4-NR					
		**TT**	10	6	61.3	23.7-NR					
		**C**	117	40	108.5	62.3-NR	**0.060**	C	2.244	**0.946-5.327**	**0.067**
		**T**	38	16	75.9	39.6-NR	0.500	-	-	-	-
	**rs4809957**	**GG**	8	2	40.3	35.0-NR	0.900	-	-	-	-
		**GA**	37	14	82.3	58.5-NR					
		**AA**	82	30	114.9	56.2-NR					
		**G**	45	16	82.3	58.5-NR	0.800	-	-	-	-
		**A**	119	44	108.5	62.1-NR	0.800	-	-	-	-
CYP2R1	**rs10741657**	**GG**	57	19	108.5	62.1-NR	0.800	-	-	-	-
		**AG**	56	21	114.9	52.0-NR		-	-	-	-
		**AA**	14	6	75.9	58.5-NR		-	-	-	-
		**G**	113	40	108.5	61.3-NR	0.900	-	-	-	-
		**A**	70	27	75.9	53.5-NR	0.600	-	-	-	-
GC	**rs7041**	**AA**	27	5	NR	NR-NR	**0.010**	AA	1	-	-
		**AC**	71	30	61.3	41.9-NR			3.514	1.359-9.088	**0.009**
		**CC**	29	11	114.9	65.0-NR			1.997	0.692-5.761	0.201
		**A**	98	35	69.3	56.2-NR	0.500	-	-	-	-
		**C**	100	41	65.5	53.5-NR	**0.020**	AA	2.914	1.149-7.393	**0.020**

HR, hazard ratio. $IC_{95\%}$, 95% confidence interval. MST, median survival time (months). NR, not reached.
