# Supplementary figures and images for: Single Nucleotide Polymorphisms in the Vitamin D Metabolic Pathway as Survival Biomarkers in Colorectal Cancer
Source: Cancers (Basel). 2023 Aug 12;15(16):4077. doi: 10.3390/cancers15164077 (PMC10452893; doi:10.3390/cancers15164077)

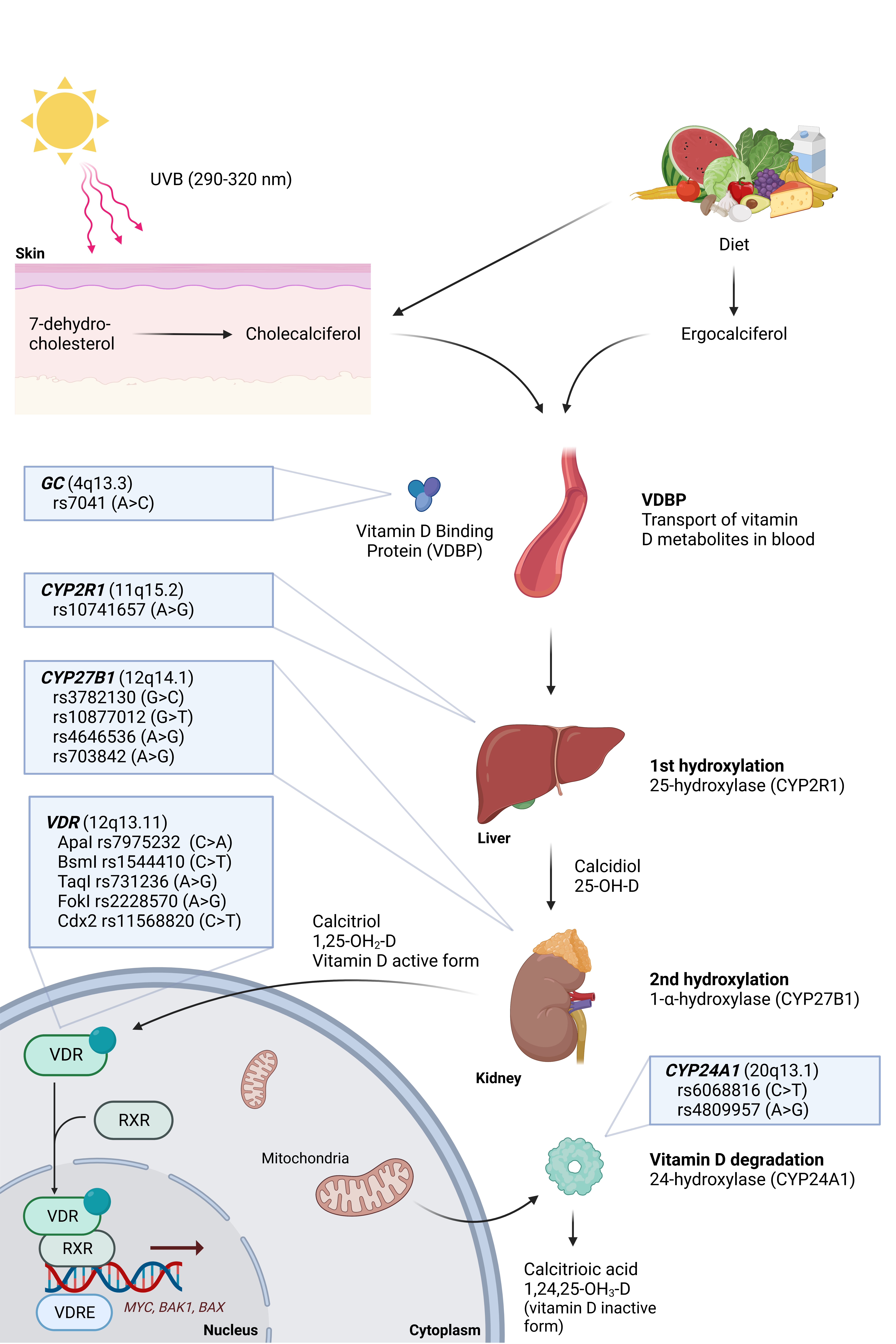

Supplement: Supplementary file 1 [file cancers-15-04077-s001.zip › Figure S1. Vitamin D Metabolism.png]

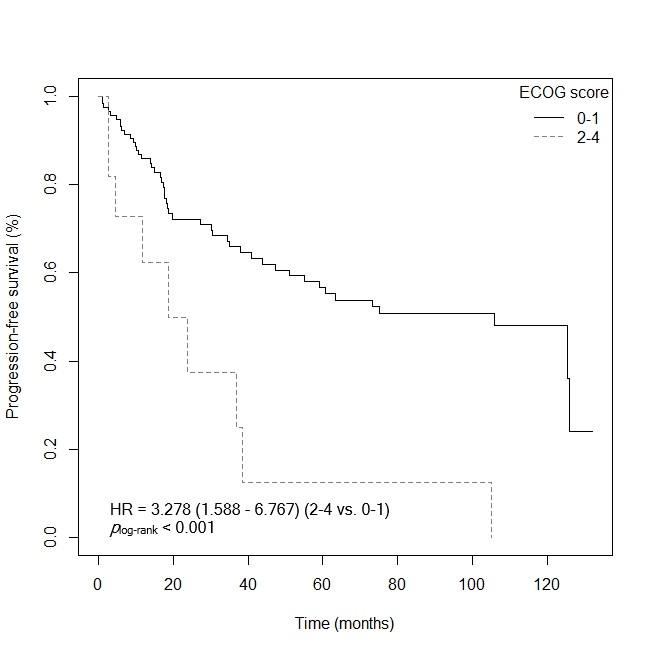

Supplement: Supplementary file 1 [file cancers-15-04077-s001.zip › Figure S10. Kaplan-Meier plot of progression-free survival curves with ECOG score in 127 patients with CRC.jpg]

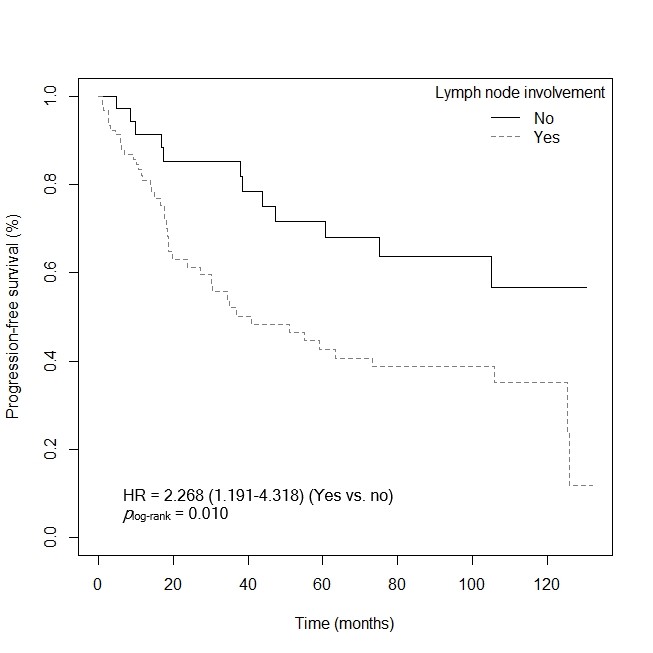

Supplement: Supplementary file 1 [file cancers-15-04077-s001.zip › Figure S11. Kaplan-Meier plot of progression-free survival curves with lymph node involvement in 127 patients with CRC.jpg]

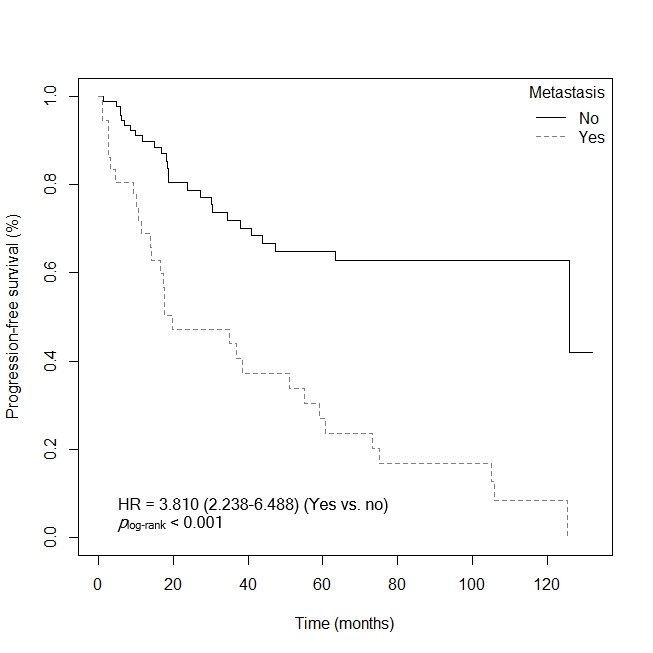

Supplement: Supplementary file 1 [file cancers-15-04077-s001.zip › Figure S12. Kaplan-Meier plot of progression-free survival curves with metastasis in 127 patients with CRC.jpg]

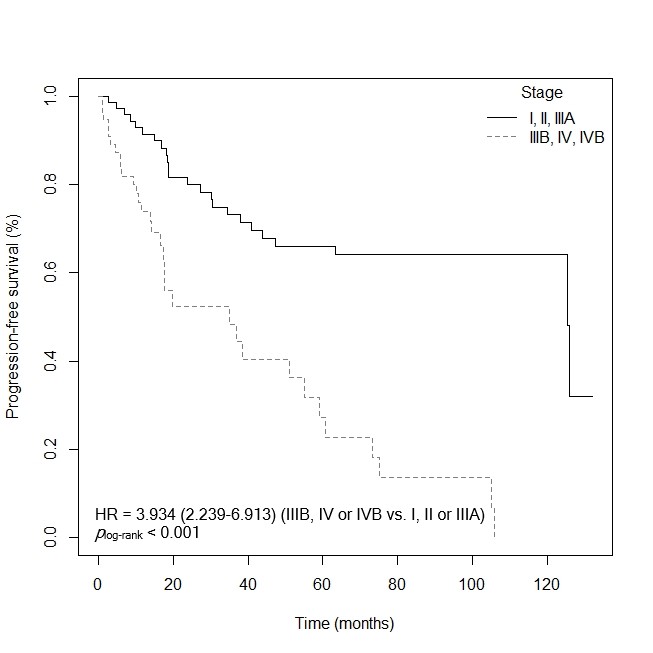

Supplement: Supplementary file 1 [file cancers-15-04077-s001.zip › Figure S13. Kaplan-Meier plot of progression-free survival curves with stage in 127 patients with CRC.jpg]

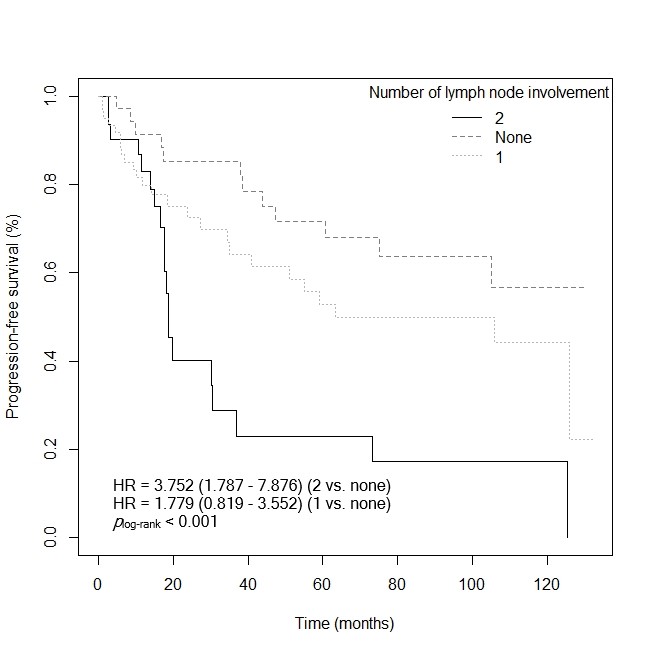

Supplement: Supplementary file 1 [file cancers-15-04077-s001.zip › Figure S14. Kaplan-Meier plot of progression-free survival curves with number of lymph node involvement in 127 patients with CRC.jpg]

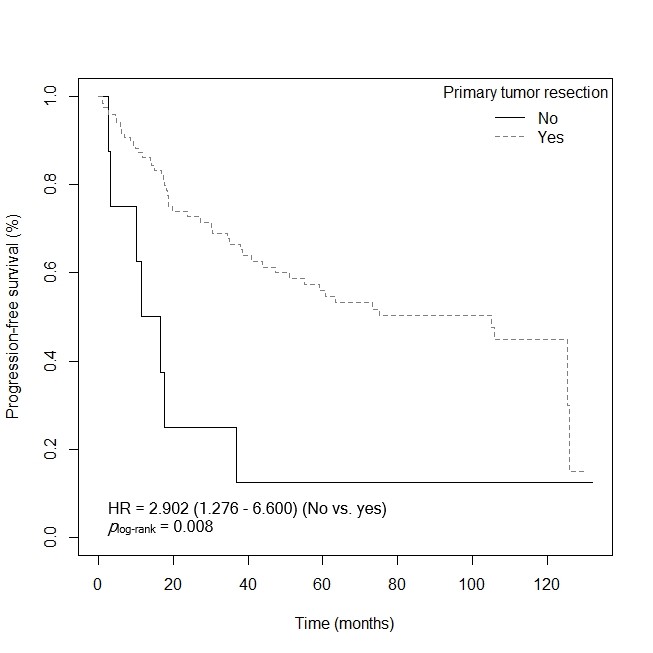

Supplement: Supplementary file 1 [file cancers-15-04077-s001.zip › Figure S15. Kaplan-Meier plot of progression-free survival curves with surgery in 127 patients with CRC.jpg]

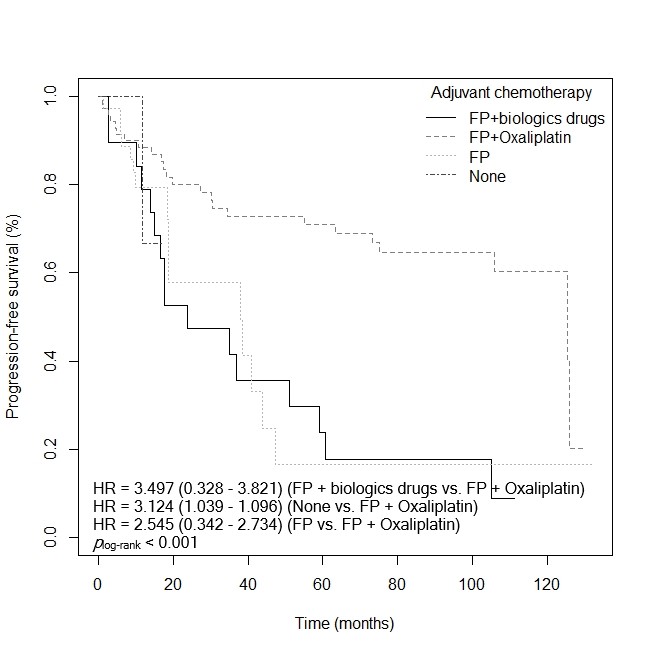

Supplement: Supplementary file 1 [file cancers-15-04077-s001.zip › Figure S16. Kaplan-Meier plot of progression-free survival curves with adjuvant chemotherapy in 127 patients with CRC.jpg]

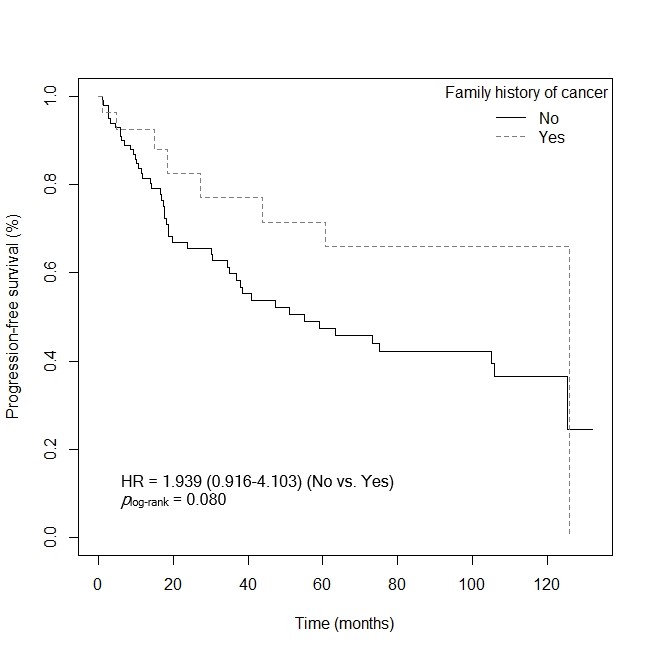

Supplement: Supplementary file 1 [file cancers-15-04077-s001.zip › Figure S17. Kaplan-Meier plot of progression-free survival curves with family history of CRC in 127 patients with CRC.jpg]

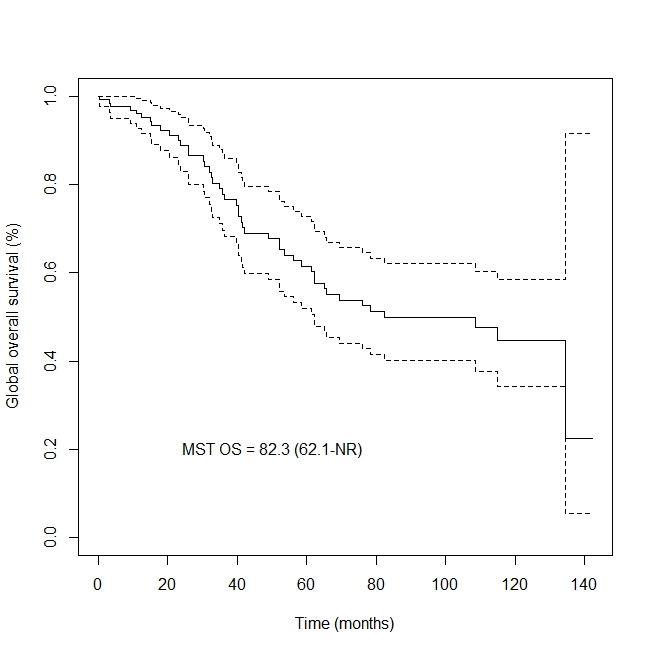

Supplement: Supplementary file 1 [file cancers-15-04077-s001.zip › Figure S2. Kaplan Meier plot OS global.jpeg]

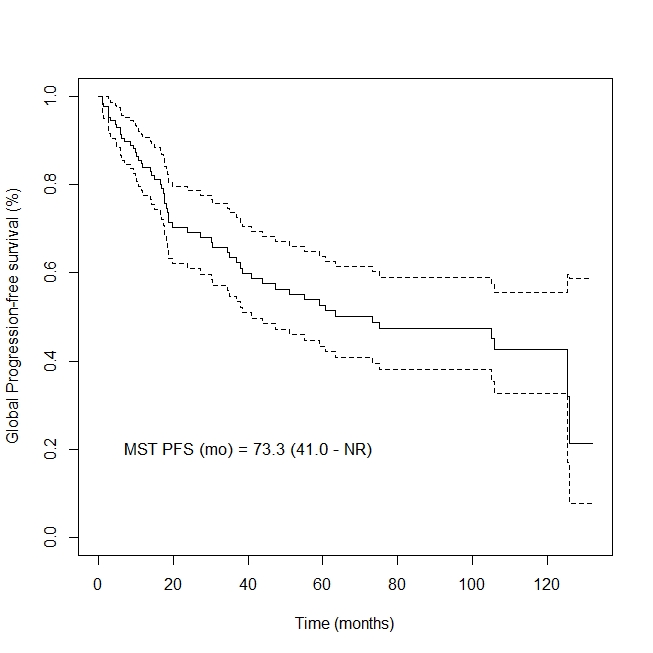

Supplement: Supplementary file 1 [file cancers-15-04077-s001.zip › Figure S3. Kaplan Meier plot PFS global.jpeg]

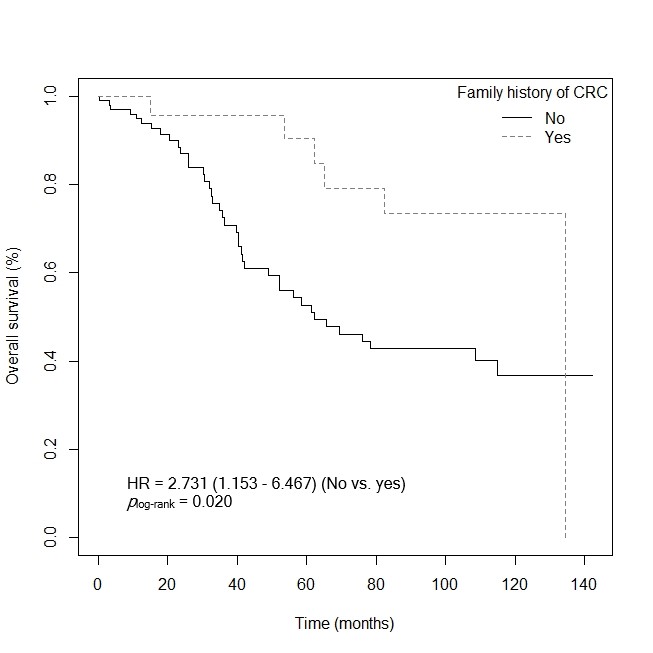

Supplement: Supplementary file 1 [file cancers-15-04077-s001.zip › Figure S4. Kaplan-Meier plot of overall survival curves with family history of CRC in 127 patients with CRC.jpg]

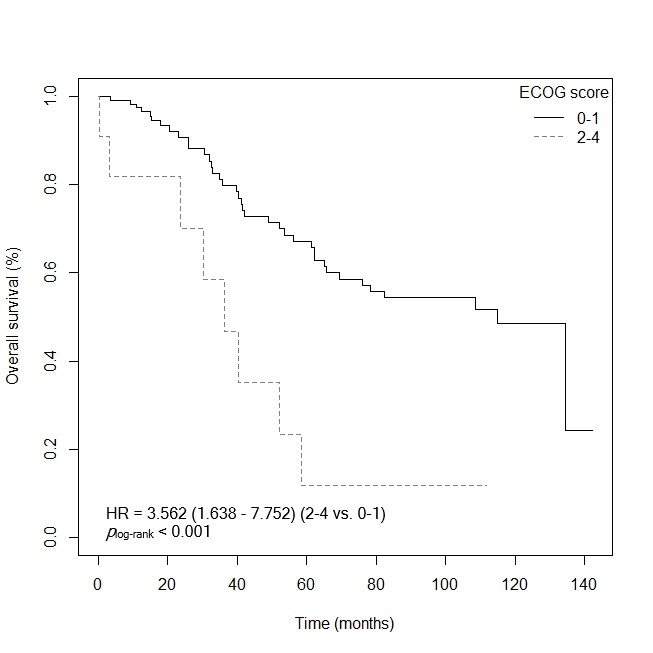

Supplement: Supplementary file 1 [file cancers-15-04077-s001.zip › Figure S5. Kaplan-Meier plot of overall survival curves with ECOG score in 127 patients with CRC.jpg]

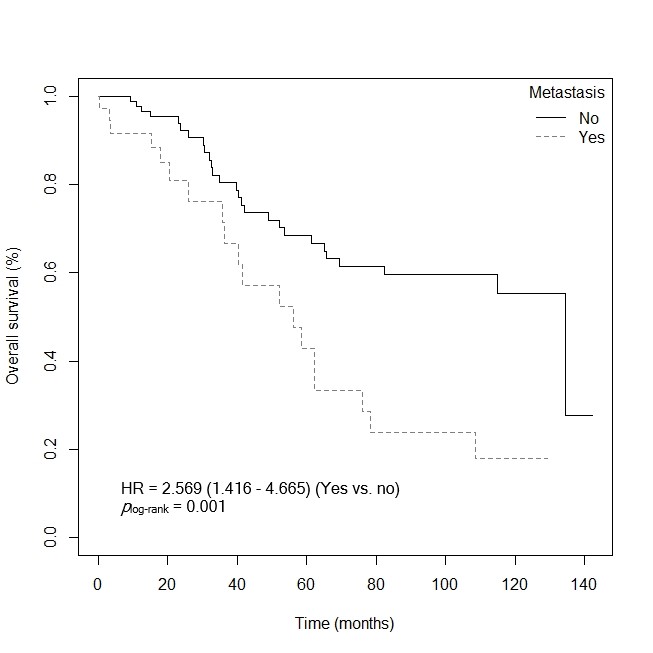

Supplement: Supplementary file 1 [file cancers-15-04077-s001.zip › Figure S6. Kaplan-Meier plot of overall survival curves with metastasis in 127 patients with CRC.jpg]

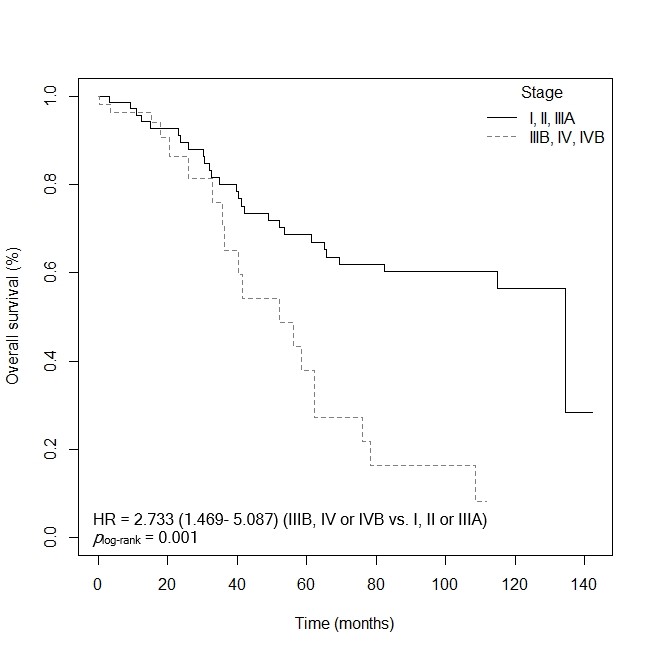

Supplement: Supplementary file 1 [file cancers-15-04077-s001.zip › Figure S7. Kaplan-Meier plot of overall survival curves with stage in 127 patients with CRC.jpg]

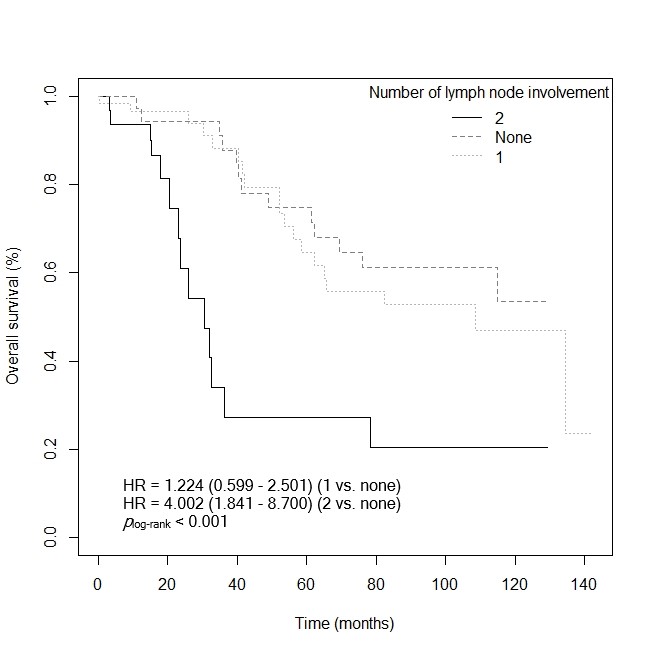

Supplement: Supplementary file 1 [file cancers-15-04077-s001.zip › Figure S8. Kaplan-Meier plot of overall survival curves with number of lymph node involvement in 127 patients with CRC.jpg]

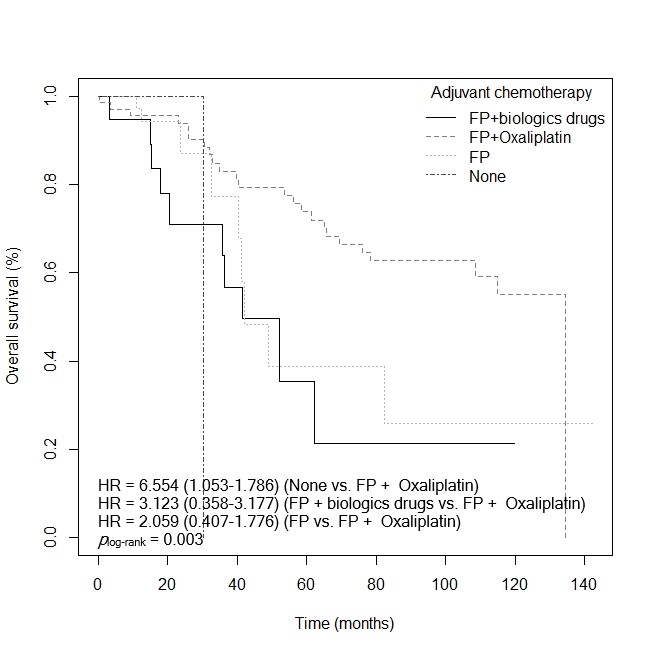

Supplement: Supplementary file 1 [file cancers-15-04077-s001.zip › Figure S9. Kaplan-Meier plot of overall survival curves with adjuvant chemotherapy in 127 patients with CRC.jpg]
